# Intracranial Large Artery Involvement in Cerebral Autosomal Dominant Arteriopathy with Subcortical Infarcts and Leukoencephalopathy: A Tale of Two Genes?

**DOI:** 10.3390/genes16080882

**Published:** 2025-07-26

**Authors:** Marialuisa Zedde, Rosario Pascarella

**Affiliations:** 1 Neurology Unit, Stroke Unit, Azienda Unità Sanitaria Locale-IRCCS di Reggio Emilia, Viale Risorgimento 80, 42123 Reggio Emilia, Italy; 2 Neuroradiology Unit, Ospedale Santa Maria della Misericordia, AULSS 5 Polesana, 45100 Rovigo, Italy

**Keywords:** Cerebral autosomal dominant arteriopathy with subcortical infarcts and leukoencephalopathy, CADASIL, intracranial stenosis, small vessel disease, large artery, MRI, angiography, *NOTCH3*, *RNF213*

## Abstract

**Background/Objectives**： Cerebral autosomal dominant arteriopathy with subcortical infarcts and leukoencephalopathy (CADASIL) is a prevalent Mendelian disorder caused by mutations in the *NOTCH3* gene, primarily impacting cerebral small blood vessels. This review aims to explore the involvement of large intracranial arteries in CADASIL, particularly focusing on the association with *RNF213* polymorphisms, especially in Asian populations. **Methods**： A comprehensive literature review was conducted to gather data on the morphological features of both small and large intracranial arteries in CADASIL, examining clinical manifestations, imaging findings, and genetic associations. **Results**： The findings indicate that while CADASIL is predominantly characterized by small vessel disease, a significant number of patients also exhibit large artery involvement, particularly Asian populations where *RNF213* polymorphisms may play a critical role. The review highlights the evidence of intracranial stenosis and the potential implications of traditional vascular risk factors, such as hypertension and diabetes mellitus, which are prevalent in these populations. **Conclusions**： The involvement of larger intracranial arteries in CADASIL underscores the complexity of the disease, suggesting that both genetic predispositions and environmental factors contribute to vascular abnormalities. Further research is needed to clarify these relationships and improve diagnostic and therapeutic strategies for CADASIL patients.

## 1. Introduction

Cerebral autosomal dominant arteriopathy with subcortical infarcts and leukoencephalopathy (CADASIL, type 1) is recognized as the most prevalent Mendelian disorder affecting the cerebral small blood vessels [1,2,3]. This condition arises from mutations in the *NOTCH3* gene, located on the short arm of chromosome 19, specifically between bands 13.2 and 13.1 [4,5]. The human *NOTCH3* gene consists of 33 exons that code for single-pass transmembrane receptors containing 2321 amino acids [5]. Most pathogenic variants occur within exons 2–24, which are associated with 34 epidermal growth factor-like repeats (EGFRs), resulting in an odd number of cysteines in a specific EGFR [3].

Microscopically, CADASIL is characterized by an abnormal buildup of the *NOTCH3* extracellular domain (NECD) around vascular smooth muscle cells (VSMCs) and pericytes [6]. The exact role of disrupted canonical Notch signaling in the disease process remains uncertain; however, the accumulation of various extracellular matrix (ECM) proteins alongside NECD has been proposed as a contributing factor to small-vessel pathology in CADASIL [7]. Despite identifying the causative gene over 25 years ago, the precise mechanism underlying the disorder is still unclear.

Historically, CADASIL was considered a rare genetic disorder, with an estimated prevalence of 1.3–4.1 per 100,000 individuals among Caucasians [8,9,10,11]. Recent extensive genomic studies, however, have revealed a significantly higher prevalence of pathogenic *NOTCH3* variants in the general population, estimated at 3.4 per 1000 individuals, with the highest risk observed among Asians [12,13]. The increased accessibility of genetic diagnosis has led to the identification of more CADASIL patients, prompting several studies to explore its clinical manifestations in order to predict its clinical trajectory. Unfortunately, establishing a significant genotype–phenotype correlation in CADASIL remains challenging due to considerable variability in disease onset and severity, even among individuals with the same mutation or from the same family [14,15,16].

Magnetic resonance imaging (MRI) of the brain is the most effective imaging modality for differential diagnoses, assessing disease severity, and predicting outcomes in CADASIL patients [17,18,19]. Characteristic MRI features can aid in differentiating CADASIL from sporadic ischemic stroke, with both the number of lacunes and the extent of brain atrophy proving useful for predicting the clinical outcomes in CADASIL patients. In situations where brain MRI is unavailable, blood biomarkers may serve as valuable tools for screening high-risk individuals, diagnosing clinically probable patients lacking genetic evidence, and predicting clinical outcomes and prognosis [20,21].

CADASIL is defined as a non-amyloid, non-arteriosclerotic arteriopathy that impacts arteries ranging from 50 to 500 μm in various areas, such as the white matter, basal ganglia, thalamus, brain stem, cerebellum, and leptomeninges [3,22]. A key pathological characteristic is the concentric thickening of the arterial wall, caused by the accumulation of granular osmiophilic material (GOM) within the media, which leads to hyaline degeneration and narrowing of the lumen [3,22]. Similar pathological alterations have been observed in other organs, including the heart, muscle, skin, liver, and spleen [23,24]. The role of large brain arteries in CADASIL remains unclear; however, the significant presence of small artery disease in CADASIL patients suggests that larger arteries might also be affected. While some research has indicated the occurrence of intracranial large artery stenosis in these patients [25,26], the impact of CADASIL on larger and medium-sized cerebral arteries is not yet fully understood. Large and small brain arteries are interconnected through shared hemodynamic pathways, creating a continuum of arterial exposure from the main large artery to its smaller branches [27]. Findings from a translational study using *Notch3* knockout mice (*Notch3*^−/−^) suggest that the absence of the *NOTCH3* gene is associated with increased tortuosity and dilation of the middle cerebral artery compared to wild-type mice, reinforcing the idea that CADASIL may also affect larger arteries [28]. Additionally, some observations, particularly in Asian patients, report intracranial large artery involvement in CADASIL patients, primarily as intracranial stenosis, often associated with the ring finger protein 213 (*RNF213*) polymorphism c.14576G > A (p.R4859K, rs112735431) [29]. *RNF213* is linked to intracranial stenosis and systemic arteriopathy in Asian populations, although its precise function remains unknown [30,31].

The aim of this review is to collect and present the morphological features of small and large intracranial artery involvement in CADASIL, starting with available reports and proposing a pathophysiological approach to this association.

## 2. Intracranial Vessel Involvement in CADASIL

The clinical manifestations of CADASIL encompass migraine with aura and recurrent ischemic attacks, which can ultimately lead to cognitive decline and dementia [32]. Symptoms generally begin to emerge in midlife, but they may present as early as 25 years of age or as late as 60 years [3,33].

### 2.1. Small Arteries Involvement

The main neuroradiological features of CADASIL are easily identified in T2-weighted MRI, showing characteristic hyperintensities in the periventricular and anterior temporal lobe white matter, often detected prior to the onset of overt clinical symptoms [34]. T1-weighted MRI can better image multiple small lacunar infarcts, which are usually located in the white matter and deep gray matter while sparing the cerebral cortex [35,36]. These imaging findings are corroborated by neuropathological studies [22,37,38]. The primary pathological feature of CADASIL is an arteriopathy marked by the degeneration of vascular smooth muscle cells (VSMCs). GOM deposits are typically found adjacent to the degenerating VSMCs in the small arteries across nearly all organs [23,24]. Post-mortem examinations generally reveal multiple small infarcts and widespread myelin loss in both white and deep gray matter [23]. The exact mechanism by which damage to small penetrating arteries results in brain infarcts in CADASIL patients remains unclear. Traditionally, it has been proposed that the walls of arterioles in the white matter thicken due to the accumulation of various extracellular matrix components, debris from degenerating VSMCs, and GOM. This fibrotic thickening is thought to reduce the luminal size and compliance of arterioles, ultimately diminishing cerebral blood flow and leading to thrombosis in the affected arterioles [32,39]. Recent research by Brulin et al. [39] has suggested an alternative pathogenic mechanism, indicating that there is no significant thickening of arteriolar and capillary walls in the skin or brain of CADASIL patients. They proposed that true arterial stenosis does not occur; rather, the loss of VSMCs weakens the walls of blood vessels, resulting in hypotonicity of the arteriolar wall, which becomes more susceptible to collapse, leading to thrombosis and localized capillary leaks [39].

An autopsy study of four Finnish CADASIL patients with the C475T (R133C) mutation examined small arteries in both gray and white matter to assess whether true stenosis of fibrotic white matter arterioles constitutes a critical pathogenic event [40]. The authors observed narrowed lumina in white matter arterioles, which had significantly thickened walls containing GOM in the tunica media. Additionally, the adventitia displayed intense immunostaining for collagen I, while α-smooth muscle actin staining in the media of thickened arteriolar walls was irregular in CADASIL patients compared to cerebrovascular controls, which showed continuous and uniform shapes. Furthermore, strong immunopositivity for the extracellular domain of *NOTCH3* was noted in the degenerating tunica media of arterioles within the white matter of CADASIL patients. Electron microscopy revealed the characteristic presence of GOM in arterioles of both gray and white matter in CADASIL brains. Interestingly, the authors conducted a morphometric analysis of all arterioles with an external diameter of 30 µm or larger and a maximum of 300 µm in a representative section of the frontal lobe. The sclerotic index was calculated as 1 (internal diameter/external diameter) [41,42]. According to the values proposed by Lammie et al. [42] for cerebral small vessel disease (cerebral arteries < 300 µm in diameter), a sclerotic index of <0.3 indicates normal arteries, 0.3 to 0.5 indicates moderate affection, and >0.5 indicates severe affection of arteries/arterioles. In the reported study, the sclerotic index increased sharply when the internal diameter of arterioles narrowed to 20 to 30 µm and the external diameter to about 130 µm. Longitudinal serial section analyses showed that the sclerotic index remained relatively constant in gray matter and superficial white matter arterioles, with only minor fluctuations. However, it sharply increased in deep white matter when the internal diameter of arterioles narrowed to 20 to 30 µm and the external diameter to about 100 µm. This study [40] demonstrated a significant increase in the wall thickness of white matter arterioles in deceased CADASIL patients, as indicated by a notably higher sclerotic index compared to controls. Importantly, the results indicated that the substantial rise in the sclerotic index of white matter arterioles did not occur until the internal diameters decreased to approximately 20 to 30 µm and the external diameters ranged from about 100 to 130 µm. This observation applies to both primary penetrating arterioles and their branches, with the increase in the sclerotic index associated with significant lumen narrowing, rather than being solely attributed to outward growth of the arteriolar wall.

Overall, these findings underscore that CADASIL is primarily a disorder affecting the arterioles within the brain parenchyma, leading to significant stenosis. Similar variations in the sclerotic index have been noted in subcortical arteriosclerotic encephalopathy (Binswanger disease), where the sclerotic index of white matter arteries in the frontal lobe was significantly elevated compared to controls, similar to the observations in CADASIL [41]. However, some researchers [43] did not find evidence of stenosis but reported dilation of penetrating arteries, with narrowed distal segments and branches of the penetrating arterioles; dilation was only observed in larger caliber segments, beyond the size at which the sclerotic index begins to increase. This observation was corroborated by the Finnish study [40], which noted dilation in penetrating arteries with larger diameters. Brulin et al. [39] analyzed brain vessels from five CADASIL cases and found no significant differences in the sclerotic index of white matter capillaries between CADASIL and control groups or differences in the sclerotic index of capillaries between gray and white matter in CADASIL. In contrast, the Finnish study presented a different interpretation of the same values reported by the authors [40]. In CADASIL, the sclerotic index was significantly higher in white matter compared to gray matter, while the opposite was true for controls. This suggests a more severe impact on white matter arterioles in CADASIL, whereas cortical arterioles appeared relatively better preserved. Despite similar degrees of VSMC degeneration and GOM deposition in both white and gray matter [40], the disparity in the sclerotic index between CADASIL patients and controls was much more pronounced in white matter than in gray matter, indicating that arterioles in these regions respond differently to the same degenerative process. The adventitial thickening suggests that vascular mesenchymal cells responsible for producing extracellular matrix react more vigorously to VSMC degeneration in CADASIL. A similar divergence between cortical and white matter arteries/arterioles is observed in cerebral amyloid angiopathy, where β-amyloid peptide deposition predominantly occurs in the walls of cortical vessels [44]. These findings align with studies on cerebral hemodynamics and support the established observation that infarctions in CADASIL are virtually absent in the cerebral cortex [24,32]. Using the MRI bolus tracking method, decreased cerebral blood flow (CBF) or regional cerebral blood volume (rCBV) has been identified in the cerebral white matter of symptomatic CADASIL patients [45,46], while cortical CBF or rCBV remains unaffected. Additionally, positron emission tomography has revealed reduced CBF in the white matter even at early stages of the disease (in patients aged 19–42), whereas cortical CBF showed no significant decrease [47]. In CADASIL, the presence of stenosis along with marked thickening and fibrosis of the vessel wall, particularly in small arteriolar resistance vessels, aligns well with other hemodynamic studies. The arterial response to acetazolamide and CO_2_ is significantly diminished [46,48], likely due to decreased compliance of the thickened, fibrotic arterioles. The stenosis of these vessels corresponds with the findings by Liebetrau et al. [49], who reported prolonged cerebral transit time, indicative of reduced blood flow velocity. Similarly, a decrease in the mean flow velocity in the middle cerebral artery has been documented [48], and van den Boom et al. found reduced baseline total CBF as measured by phase contrast magnetic resonance imaging [50]. The increased resistance at the small arteriolar level may also explain the dilation of the penetrating medullary arteries reported by Okeda et al. [43].

It remains unclear how the early destruction of VSMCs and the accumulation of connective tissue in cerebral arterioles reach a threshold that leads to significant reductions in blood flow, resulting in ischemic symptoms. Additionally, transient ischemic attacks and migraines are likely induced by reversible phenomena. One speculative hypothesis is that the affected VSMCs in these vasoregulatory arterioles may respond with chronic contraction, which reduces arteriolar luminal size and contributes to further VSMC degeneration. In CADASIL, the medullary arteries of the frontal lobe may demonstrate a complete loss of medial smooth muscle cells along their entire length, accompanied by severe adventitial fibrosis that extends into the white matter [43]. Although complete occlusion is not observed, the long penetrating arterioles and their branches supplying subcortical structures are stenosed, with their walls thickened by fibrosis. This condition contributes to an increased incidence of infarcts and primary ischemic damage within the white matter [40]. Arteriolosclerotic changes impede the elasticity necessary for these vessels to properly dilate and constrict in response to fluctuations in systemic blood pressure or autoregulation, leading to altered blood flow responses and hemodynamic changes that affect tissue perfusion. Depending on the size of the microvessels, these perfusion alterations can result in lacunar infarcts (cystic lesions typically <1 cm) and microinfarcts. Deep cerebral structures and white matter are particularly susceptible, as these vessels function as end-arteries with minimal anastomoses. However, certain intrinsic arteriolar systems may exhibit differential susceptibility to these changes.

**Figure 1:**
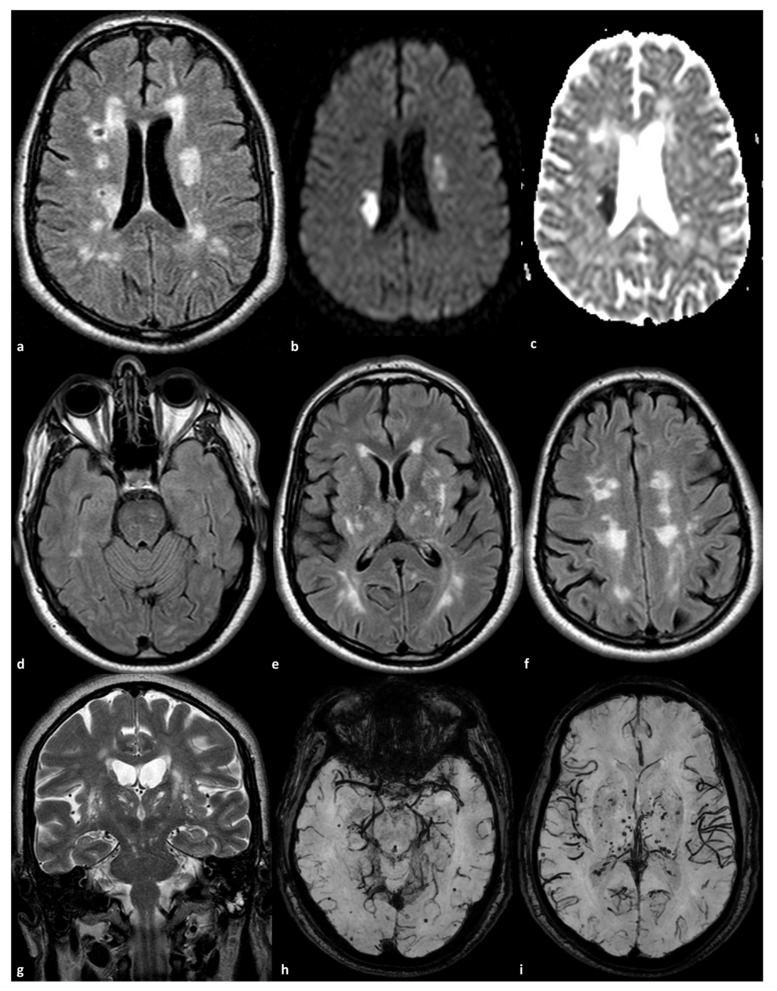
Brain MRI of a 56-year-old man with a homozygous *NOTCH3* mutation p.R1231C (Ex22) and a past history of arterial hypertension. The diagnosis was performed after admission to the Stroke Unit due to acute stroke (panel (**a**): axial FLAIR; panel (**b**): DWI; panel (**c**): ADC map) with a dual DWI-positive subcortical lesion in the corona radiata of right and left hemispheres. The right stroke was the most recent one. The remaining brain in FLAIR (panels (**d**–**f**)) showed symmetrical WMHs sparing the anterior temporal pole and involving the external capsule, the basal ganglia, the subcortical white matter in the occipital lobes, and the centrum semiovale. EPVSs in the basal ganglia are evident in T2WI in coronal view (panel (**g**)). Several subcortical lobar (panel (**h**)) and deep (panel (**i**)) cerebral microbleeds are present in susceptibility-weighted imaging.

More recently, 7T MRI has enhanced the ability to investigate small vessels, particularly lenticulostriate arteries (LSAs) in three-dimensional time-of-flight MR angiographic study [51]. This study showed no differences in the luminal diameters of the LSAs between CADASIL patients and control subjects. The lumina of the lenticulostriate arteries were not associated with lacunar infarct load in the basal ganglia area or with basal ganglia hyperintensities [52]. Conversely, a pathological study reported that arteriolar lumina in the lenticular nuclei were larger than those in the white matter and also larger than in cortical grey matter, which rarely develops infarcts [52]. The MRI study [51] included 46 CADASIL patients and 46 age- and sex-matched healthy controls. Participants underwent 7.0-T MRI, which encompassed T1-weighted imaging, 3D time-of-flight MRA for LSAs, T2-weighted FLAIR for white matter hyperintensities (WMHs) and lacunar infarctions (LIs), and susceptibility-weighted imaging for cerebral microbleeds (CMBs). The number and length of LSAs, the proportion of discontinuous LSAs, and clinical assessments were recorded. CADASIL patients had significantly fewer LSA branches compared to controls (mean 10.72 vs. 12.22; *p* = 0.011). A higher proportion of discontinuous LSAs was found in CADASIL patients (median 7.29 vs. 0; *p* = 0.047). No significant difference was observed in the total length of LSAs between the two groups, and no significant associations were found between LSA measurements and basal ganglia lesion load. This indicates that changes in the basal ganglia may arise from mechanisms other than anatomical narrowing of the vessels, such as hemodynamic abnormalities or dysfunction of the blood–brain barrier. An example of small vessel involvement in CADASIL is illustrated in Figure 1.

Additionally, another source of WMHs on MRI in CADASIL patients is the myelin degeneration surrounding enlarged perivascular spaces (EPVSs), as demonstrated in a pathological study focused on the anterior temporal lobes [53]. This study involved post-mortem examination of temporal pole tissue samples from nine CADASIL patients, eight subjects with sporadic subcortical ischemic vascular dementia, five young controls, and five older controls. A combination of histological techniques, including luxol fast blue-stained serial sections and immunostaining methods, was employed to assess perivascular space (PVS) extent, myelin integrity (myelin index), damage within the white matter (degraded myelin basic protein), and arteriosclerosis (sclerotic index). The CADASIL cases exhibited significantly larger PVS areas compared to controls, with markedly increased mean and total areas of PVS in CADASIL subjects, indicating pronounced pathological alterations. The myelin index was significantly reduced in CADASIL samples compared to those with SIVD and controls, and there was increased immunoreactivity for degraded myelin basic protein in CADASIL, suggesting extensive myelin degeneration.

A 4.5-fold increase in the number of basophilic (hyalinized) vessels in CADASIL patients, along with a 57% increase in sclerotic index values compared to young controls, indicates significant arteriopathic changes in the cerebral microvessels associated with PVS. These findings suggest that the WMHs observed on the MRI in CADASIL patients are attributed to enlarged PVS and myelin degeneration rather than being caused by lacunar infarcts. This observation is consistent with the fact that older patients with subcortical vascular dementia do not exhibit similar MR hypersignals in the temporal pole, suggesting that the unique anatomy and vascularization of the temporal pole may contribute to the prevalence of these changes.

### 2.2. Large Artery Involvement

More recently, individual case reports and some case-control studies, primarily focusing on the Asian population, have raised the possibility of rarer involvement of intracranial large vessels in CADASIL patients, suggesting several hypotheses about the causal or casual associations of these findings [25,26,29,54]. Interestingly, both stenosing and dilatative patterns have been reported.

As noted in previous studies [28], the decline in vascular function with age can lead to various pathologies, including stroke and chronic conditions, like cerebral hypoperfusion, ultimately culminating in cognitive impairments and dementia. Research utilizing *NOTCH3*-deficient mice alongside their wild-type counterparts has assessed the effects of *NOTCH3* loss on vascular function and neurodegeneration. Single-cell RNA sequencing was employed to examine transcriptional changes in VSMCs and pericytes across different ages, while MRI and micro-CT imaging were used to visualize vascular changes and assess cerebral blood flow in live animals. The analysis revealed a downregulation of contractile proteins and upregulation of extracellular matrix components in aged VSMCs from *NOTCH3*-null mice, indicating a switch from a contractile to a synthetic phenotype. Structural assessments demonstrated that aged *NOTCH3*-deficient mice exhibited significant vascular abnormalities, such as tortuosity, vascular enlargement, and microaneurysms. Notably, the enlargement of medium-sized vessels, like the middle cerebral artery, was found only as the artery ascended on the lateral aspects of the brain, suggesting a topological association with areas requiring increased contractile strength. In these regions, the vessel showed a 3- to 4-fold increase in volume and increased tortuosity. Secondary branches of the middle cerebral artery were characterized by beading (dilations and constrictions) along their course in aged *NOTCH3*-null mice, unlike in wild-type or heterozygous littermates. The development of microaneurysms was associated with disorganization or absence of smooth muscle cell coverage, and these changes were age-dependent, appearing only after 6 months of age and progressing over time. Importantly, while reductions in VSMC coverage were noted in other resistance arteries in aged mice, the presence of microaneurysms and beaded vessels was confined to the brain. Compromised vascular contractility was linked to impaired glymphatic function, as evidenced by reduced clearance of injected tracers in *NOTCH3*-null mice. The study highlights a significant decline in *NOTCH3* signaling in both murine and human brain vessels as a result of aging, associated with alterations in VSMCs that impact their contractile functions. Single-cell RNA sequencing identified changes in gene expression profiles in aged VSMCs, indicating a loss of contractility and an increase in extracellular matrix components, contributing to vascular dysfunction.

In an autopsy series from Japan [55], a previously autopsied case was reexamined [56]. The patient had experienced migraine with aura since age 10 and developed emotional instability, character changes, and gait disturbances at age 63, followed by disorientation, urinary incontinence, pseudobulbar palsy, and dementia. He passed away from pneumonia at age 75. His blood pressure, serum cholesterol, triglycerides, and glucose levels were normal. Typical MRI changes were noted, and his father and grandfather had similar conditions. The autopsy revealed numerous infarcts predominantly in the cerebral white matter and basal ganglia. Moderate atherosclerosis was observed in the basilar artery, along with tiny atherosclerotic foci in the internal carotid artery; anterior, middle, and posterior cerebral arteries; and vertebral artery. Arteriolar walls displayed degeneration with thickening and occasional splitting in the cerebral cortex, white matter, and meninges. While some arterioles exhibited lumen narrowing, occlusion was rarely observed. The most characteristic change was in the arteriolar media, where eosinophilic PAS-positive granular materials were deposited, showing positive immunoreactivity for complement staining but negative for IgG and h-amyloid. Electron microscopy revealed that these deposits were composed of round, oval, or amorphous masses of electron-dense fine granules.

**Figure 2:**
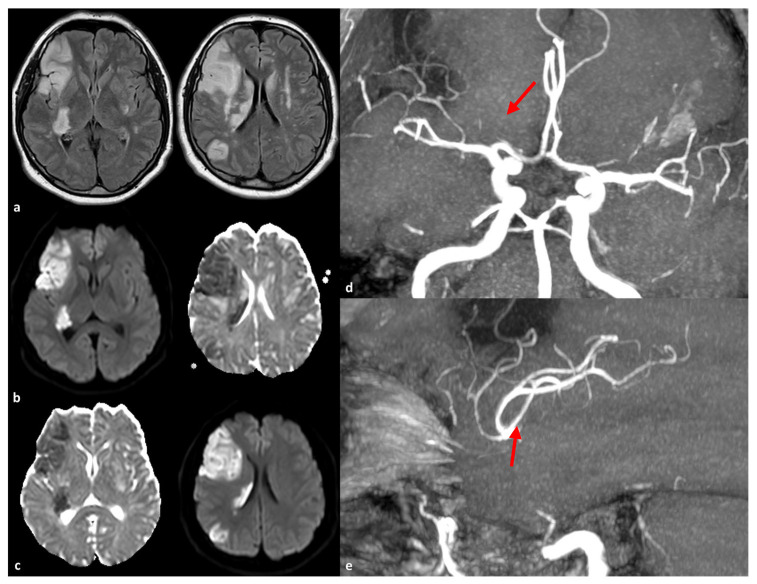
Brain MRI of a patient with a heterozygous *NOTCH3* new frame-shift mutation frame-shift p.Cys1344Leufs (Ex24). The diagnosis was performed after admission to the Stroke Unit due to acute stroke in the right MCA territory (panel (**a**): axial FLAIR; panel (**b**): DWI; panel (**c**): ADC map) with multiple DWI-positive territorial lesions. The contralateral hemisphere showed symmetrical WMHs in the deep subcortical white matter (head of the caudate nucleus, external capsule, and corona radiata). On MR angiography (time of flight), reconstructed using maximum intensity projections/multiplanar reconstruction (MIP/MPR) protocol, a residual thinning of the frontal-opercular right MCA branch is evident (panels (**d**) and (**e**), red arrow).

The initial reports of large artery involvement in CADASIL patients primarily referred to the territorial rather than lacunar patterns of ischemic stroke [25,57], suggesting that pathological involvement may extend beyond small cerebral vessels. A previous report [58] was less convincing, as it showed normal DSA, while MRA indicated abnormalities in intracranial arteries. Nonetheless, the prevalence, infarct patterns, and locations of relevant arterial diseases remain unclear. Some investigators remain skeptical about whether infarcts associated with large artery disease are manifestations of CADASIL [3]. Choi et al. [57] were the first to report large intracranial artery involvement in 13 CADASIL patients using MR angiography in 12 out of 13 cases and DSA in one. Five patients (38%) displayed stenosis: three in the middle cerebral artery, one in the vertebral artery, and one in the internal carotid artery. The stenosis persisted on follow-up angiograms in two patients. All patients exhibited diffuse leukoaraiosis and multiple lacunes in bilateral subcortical white matter and basal ganglia, consistent with cerebral small vessel disease. Two patients had additional territorial middle cerebral artery (MCA) infarcts on MRI. One of these did not have obvious angiographic abnormalities. Two years later, when she developed left hemiparesis, stenosis of the M1 portion of the right MCA was found. Diffusion-weighted MRI at this time showed scattered ischemic lesions in the right MCA territory, and follow-up angiograms four months later confirmed persistent MCA stenosis. Notably, 40% of patients with angiographic abnormalities had at least one vascular risk factor, suggesting that the concomitant presence of atherosclerosis may be causal. However, the other three patients did not present any risk factors, and there were no significant differences In risk factors between patients with angiographic abnormalities and those without. Consequently, the authors proposed the occasional involvement of large vessels in CADASIL, similar to the pathological demonstration in arterioles [40]. An autopsy study reported GOM deposition with relatively preserved vascular smooth muscle cells in the aorta, carotid, and renal arteries [23]. Atherosclerotic changes were noted in the basilar artery, ICA, and the anterior, middle, and posterior cerebral arteries in Japanese CADASIL patients without vascular risk factors [55]. Thus, abnormalities in large cerebral arteries may represent accelerated atherosclerosis in conjunction with endothelial cell damage associated with GOM deposition, although the specific triggers for large vessel diseases in particular patients remain unknown. An example of a large territorial stroke in a patient with CADASIL is illustrated in Figure 2.

A few years later, the same authors [25] updated the description of large artery involvement in 73 Korean CADASIL patients examined via MRI and MRA. Most patients (65/73, 90.3%) had the same R544C genotype, which causes an amino acid change from cysteine to arginine. A total of 40 episodes of cerebral infarction were confirmed in 31 patients, with a mean age of onset of 58.8 ± 11.4 years (range, 38–76 years). Twelve patients (16.9%) exhibited intracranial stenosis, with five of these being symptomatic. This included six cases in the middle cerebral artery, four in the posterior cerebral artery, four in the basilar artery, two in the intracranial ICA, one in the anterior cerebral artery, and one in the intracranial vertebral artery. The frequency of hypertension, diabetes mellitus, and smoking did not differ between patients with and without intracranial stenosis. However, patients in the intracranial stenosis group were significantly older (68.3 ± 12.1 years vs. 61.5 ± 10.8 years; *p* = 0.024). Two patients exhibited significant proximal ICA stenosis, with one undergoing stent insertion. The presence of intracranial stenosis was independently associated with poor clinical outcomes.

General considerations about the prevalence of intracranial atherosclerosis in Asian patients reveal that intracranial stenosis is commonly found in this population and is linked to 30% of ischemic strokes among the Chinese population [59,60]. The prevalence is also notably high in the Korean population (24.5%; mean age of onset, 58.1 years) and is significantly associated with advanced age, hypertension, and diabetes mellitus [61]. In the reported CADASIL cohort [25], 51.4% of patients had a history of hypertension, a significantly higher prevalence than observed in European CADASIL patients (6.6–20%) [62]. This elevated hypertension prevalence in our study may result from the advanced age of participants and the high prevalence of hypertension in the general Korean population.

A study involving a Korean cohort of CADASIL patients [26] examined 49 consecutive symptomatic individuals with genetically confirmed CADASIL, of whom 34 patients (69.4%) had mutations affecting cysteine. MRI scans identified infarctions occurring within the territory of relevant large cerebral arteries. Out of the 49 patients, 23 were found to have cerebral infarction, with seven of these cases linked to intracranial large artery disease (stenosis or occlusion). Specifically, in these seven patients, infarcts were located in the anterior cerebral artery (ACA) territory for four patients, the MCA territory for two patients, and the anterior inferior cerebellar artery (AICA) territory for two patients. Additionally, five other patients had asymptomatic stenosis: one in the left posterior cerebral artery (PCA), one in the right MCA, one in the right distal vertebral artery, and two in the right proximal ICA. Notably, one patient with an AICA territory infarction due to AICA occlusion later developed a new infarction in the right MCA territory when new MCA stenosis appeared. None of the seven patients with symptomatic intracranial stenosis had hypertension or diabetes, nor did they show any embolic sources from proximal arteries or the heart. Five patients underwent follow-up magnetic resonance angiography (MRA) after 2 to 22 months (mean, 13.8 months), and stenosis or occlusion persisted in all cases; three patients exhibited aggravated stenosis. No differences were found between patients with or without territorial infarction regarding vascular risk factors, microbleeds, white matter changes, or cysteine mutations.

In a separate Chinese cohort, five out of 19 cases with *NOTCH3* mutations presented with intracranial atherosclerosis, all associated with hypercholesterolemia [63]. One patient experienced a cerebral infarction due to occlusion of the left middle cerebral artery (M2 segment). Importantly, patients with large-vessel infarctions or significant major-vessel stenosis were often excluded from earlier CADASIL studies [64]. Another Chinese study [65] explored the characteristics of large arteries in CADASIL patients and their connection to cerebral small vessel disease. The authors analyzed data from 37 consecutive CADASIL patients in comparison with controls from a cohort with sporadic small vessel disease. Both groups underwent MRI and MRA at 3.0 T. Among the 37 CADASIL patients, 28 (75.7%) displayed abnormalities in intracranial large arteries, with 18 patients (48.6%) showing congenital variations and 17 (45.9%) presenting with other abnormalities; 8 out of 37 (21.6%) had intracranial stenosis. Notably, patients with severe stenosis of the MCA or ICA were more likely to exhibit an asymmetric distribution of white matter hyperintensities (WMHs). Typically, the WMH distribution is symmetric in CADASIL [3], but some cases from this Chinese study demonstrated asymmetric WMH patterns, categorized into left–right and anterior–posterior distributions. The unilateral dominant WMH pattern was found in the anterior circulation artery supply area and was associated with severe stenosis of the MCA or ICA, whereas the posterior bilateral ventricular dominant WMH pattern was mainly located in the posterior circulation supply area and often coincided with vertebral artery hypoplasia. These findings suggest that CADASIL patients may present a variety of abnormalities in intracranial large arteries that could influence the progression of microangiopathy. The study highlights the significance of evaluating large vessels in CADASIL, as their abnormalities may correlate with the distribution of WMHs.

In the reported studies, patients with infarctions linked to large artery disease represented 14.3% of all symptomatic CADASIL patients and 30.4% of those with ischemic stroke [26]. Furthermore, symptomatic cerebral artery stenosis was found in 6.8% of CADASIL patients (five out of 73) [25]. In a Chinese cohort, symptomatic cerebral arterial stenosis was identified in 5 out of 18 (26%) CADASIL patients [66]. Additionally, an autopsy study from Japan revealed arterial stenosis in the basilar artery (BA), ICA, ACA, MCA, and PCA in a CADASIL patient [53]. Notably, arterial stenosis was typically observed in smaller vessels (such as the ACA, AICA, and distal M1 or M2 segments of the MCA) rather than in the usual locations for intracranial atherosclerosis (like the proximal MCA, proximal PCA, and basilar artery) [64].

Thus, while small artery disease is a primary manifestation of CADASIL, large artery disease may also occur. It could be argued that large arterial disease is simply an epiphenomenon unrelated to the CADASIL genetic mutation. However, no significant differences in age or risk factors were observed between patients with and without infarctions associated with large artery disease. The exact nature of vascular stenosis and occlusion remains speculative without pathological evaluation. Partial embolic occlusion or vascular spasms seem unlikely, as no patients had identifiable embolic sources, and vascular lesions were persistent or worsened in those who underwent follow-up imaging. Although pathological changes are primarily known to affect small cerebral arteries, a report from Japan indicated the presence of “atherosclerotic changes” in multiple cerebral arteries [54].

Another interesting finding emerged from individual reports of fusiform dilation in the intracranial arteries of CADASIL patients [67,68]. Lopez-Navarro et al. [53] conducted a retrospective case-control study involving 37 CADASIL patients and 104 randomly selected controls with acute lacunar strokes, focusing on the diameters of the BA and ICA using T2-weighted MRI. Z-scores of the arteries were calculated to create a Brain Arterial Remodeling (BAR) score. CADASIL patients were found to be less likely to be Hispanic/Latino (*p* < 0.001), hypertensive (*p* < 0.001), or current smokers (*p* = 0.02), but more likely to have a prior stroke (*p* < 0.001) compared to controls. In adjusted models, CADASIL patients exhibited larger diameters of the BA than controls (*p* = 0.002), with no significant differences in the right and left ICA diameters (*p* = 0.73, *p* = 0.88). There was a statistical trend indicating higher cervical ICA tortuosity in CADASIL patients compared to controls (*p* = 0.08). These results suggest that the CADASIL phenotype extends beyond small brain arteries to include their parent large arteries. However, it remains uncertain whether the changes observed in the basilar and potentially cervical arteries reflect in situ pathology, such as degeneration of the media, which serves as the primary mechanical barrier against flow-related dilation [69,70]. Media degeneration has been linked to various connective tissue disorders and arteriopathies that manifest with dilated and/or tortuous cerebral arteries, including dolichoectasia, which occurs in populations without CADASIL [71,72,73,74]. In CADASIL, smooth muscle cells are eventually replaced by GOM. Degeneration of the media in larger arteries, more so than in smaller ones, may predispose them to flow-mediated outward remodeling [39]. Alternatively, progressive neurodegeneration could reduce neurotrophic support to the neurovascular unit, leading to arteriolar dilation and, ultimately, flow-induced outward remodeling [75]. The BA is particularly unique due to its origin from the convergence of two vertebral arteries, creating flow dynamics that may predispose it to dilation [76,77]. Pathologically, the basilar artery exhibits more evidence of outward remodeling compared to arteries in the anterior circulation. The authors acknowledged the limitations of this study, which include an imbalance between groups due to unmeasured residual confounding factors related to race/ethnicity, variability in imaging modalities, and the lack of correction for head size when evaluating brain arterial diameters [78,79]. Nonetheless, the clinical implications of larger basilar artery diameters warrant further exploration in a larger sample with standardized MRI imaging acquisition and a more thoroughly characterized clinical and cognitive status.

## 3. The Role of *RNF213* Polymorphisms

The *ring finger protein 213* gene (*RNF213*; NM_001256071.2) encodes a 590 kDa protein that incorporates a RING finger domain with E3 ubiquitin-protein ligase activity and two ATPase-associated domains. *RNF213* is implicated in angiogenesis and vascular inflammation, as evidenced by both in vitro and in vivo studies, yet its precise physiological functions remain ambiguous [80]. In particular, *RNF213* is expressed ubiquitously throughout the body, localizes to the surface of intracellular fat droplets, and contributes to vascular homeostasis by promoting lipolysis. *RNF213* is also involved in the regulation of endoplasmic reticulum stress response, and it is upregulated by lipopolysaccharide, tumor necrosis factor-α (TNF-α), and interferons. However, little is known about the ubiquitination substrates and molecular cascade of *RNF213* that may be involved in abnormal angiogenesis [80].

The significance of this gene as a locus for vasculopathy susceptibility was first demonstrated in 2011 through genome-wide linkage analysis in a cohort of Japanese families with moyamoya disease, where heterozygosity for the *RNF213* p.Arg4810Lys (c.14429G > A, rs112735431) polymorphism was significantly associated with the disease [81]. Moyamoya disease is characterized as a progressive, non-atherosclerotic, non-inflammatory steno-occlusive disorder that leads to the formation of compensatory collateral networks, hence its name [82].

Following the initial discovery, a genome-wide association study in Japanese patients with moyamoya disease revealed that 95% of familial and 73% of non-familial patients carried the *RNF213* p.Arg4810Lys variant, which conferred an odds ratio of 190.8 for the disease [83]. Similar associations were confirmed in the Korean and Chinese cohorts of moyamoya patients [84,85], although the allele frequency for the *RNF213* p.Arg4810Lys variant was lower in Chinese patients compared to their Japanese and Korean counterparts. Additionally, another variant, *RNF213* p.Ala5021Val, has been identified as a susceptibility factor for moyamoya disease in Chinese patients [86]. In larger cohorts, the *RNF213* (c.14576G > A) mutation was reported in 69.9% to 85.4% of moyamoya disease cases [87,88,89]. In non-Asian and particularly Caucasian patients, the *RNF213* p.Arg4810Lys variant is exceedingly rare, with several distinct pathogenic variants identified in this population [86]. These variants show a lower odds ratio (2.24) and a strong association with moyamoya disease primarily in familial cases [90]. Furthermore, the *RNF213* c.14576G > A variant has been linked to other vascular diseases, with initial associations made with pulmonary artery stenosis (peripheral pulmonary artery stenosis with pulmonary hypertension) and coronary artery conditions [91]. These conditions can sometimes co-occur with moyamoya disease [90,92], particularly in the carriers of the homozygous *RNF213* p.Arg4810Lys mutation. Over time, the concept of *RNF213*-associated vasculopathy has evolved [93]. It has been proposed that the heterozygous R4810K variant results in classical moyamoya disease, whereas the same variant in a homozygous state is associated with moyamoya disease along with systemic vascular disease in a gene dosage-dependent manner [90]. Homozygous patients often present with diffuse narrowing of the aorta and iliofemoral arteries, as well as stenosis of renal, celiac, or peripheral pulmonary arteries, with or without moyamoya disease [94]. In contrast, heterozygous patients typically remain asymptomatic or present only with isolated moyamoya disease. This association indicates a high penetrance of systemic vasculopathy in homozygous patients and a low penetrance of moyamoya disease in heterozygous individuals.

The *RNF213* (c.14576G > A) variant is implicated in 21% to 23.2% of cases of intracranial internal carotid artery stenosis, but it has not been associated with vertebral artery stenosis [88,95]. This variant is notably prevalent among East Asians without intracranial disease, with allele frequencies reported as 2.8% in Japanese patients, 2.5% in Korean patients, and 1.1% in Chinese patients, while it is rarely found in Western Caucasians [88,96]. Interestingly, the frequency of the *RNF213* (c.14576G > A) variant in Japanese individuals with cerebral aneurysms is lower than in those with intracranial stenosis, often comparable to that in control subjects without vascular lesions, ranging from 0% to 2.1% [97]. Additionally, the *RNF213* variants, such as p.Arg2438Cys and p.Ala2826Thr, have been identified in French-Canadian patients with intracranial aneurysms. However, limited studies have investigated the relationship between *RNF213* and intracranial aneurysms, leaving various aspects, including the site, morphology (saccular, fusiform, or dissected), and clinical characteristics of these aneurysms, unclear [98]. The etiology of intracranial aneurysms can vary significantly based on their origin within the intracranial vessels. This variation is accompanied by differences in frequency between men and women, as well as differing rupture rates, highlighting the need for a nuanced assessment approach [99,100].

Yeung et al. [29] aimed to determine whether *RNF213* is associated with intracranial stenosis in CADASIL by genotyping rs112735431 for 124 CADASIL patients. They found a carrier rate of c.14576G > A in CADASIL patients with intracranial stenosis (4/17; 23.5%), significantly higher than those without intracranial stenosis (2/107; 1.9%) (*p* = 0.0032). Moreover, among patients with intracranial stenosis, the frequency of territorial infarction was significantly higher in c.14576G > A carriers (75.0%) compared to non-carriers (20.0%) (*p* = 0.0410). The rate of ≥50% stenosis or occlusion tended to be higher in c.14576G > A carriers (4/4; 100%) than in non-carriers (6/13; 46.2%) (*p* = 0.1029). Among the four carriers, three had MCA stenosis, and one had multiple stenoses (left ICA + right PCA + BA). During follow-up, one *RNF213* variant carrier exhibited progressive MCA stenosis, ultimately leading to complete occlusion of the artery, while another carrier experienced a territorial infarction due to complete MCA occlusion. The key finding of this study is that the c.14576G > A variant is a susceptibility factor for intracranial artery stenosis in CADASIL patients. Notably, 21.9% of patients with sporadic intracranial stenosis in Japan exhibited the c.14576G > A variant [5], which is comparable to the 23.5% observed in CADASIL patients with intracranial stenosis. In a control population from Japan, 2.6% (10 out of 384) were identified as carriers of the c.14576G > A variant [81], similar to the 1.9% found in the CADASIL cohort without intracranial stenosis. These findings suggest that the association of *RNF213* with susceptibility to intracranial stenosis is consistent across different populations.

The aforementioned study [29] found that 13.7% (17 out of 124) of Japanese CADASIL patients had intracranial stenosis. This rate is lower than that reported in Korean patients, where the prevalence of ICAS was 38% (5 out of 13) [56] and 24.5% (12 out of 49) [26]. Interestingly, the frequencies of ICAS in non-stroke patients are reported as 14.7% in Japanese individuals [101] and 24.5% in Korean individuals [66]. The similar intracranial stenosis frequencies observed between CADASIL patients and non-stroke patients of each ethnicity suggest that CADASIL mutations may not significantly influence the occurrence of intracranial stenosis. However, inconsistencies in the definition of intracranial stenosis across various studies complicate the comparison of ICAS prevalence and *RNF213* carrier frequency in intracranial stenosis among different reports (Table 1).

Recently, an individual case report described a patient with CADASIL who also exhibited *RNF213*-related vasculopathy [102]. This patient experienced early-onset recurrent strokes and progressive intracranial artery stenosis, carrying both heterozygous *NOTCH3* p.Cys1250Arg and *RNF213* p.Arg4810Lys variants. Additionally, he had mild dyslipidemia and type 2 diabetes mellitus. The first stroke occurred at age 62 (affecting the right thalamus), followed by a second stroke at age 63 (an isolated acute infarct in the left temporal pole with ipsilateral middle cerebral artery stenosis confirmed by digital subtraction angiography), and a third stroke at age 66 (resulting in a pontine lesion). Over four years of monitoring through longitudinal MR angiography, progressive stenosis of the left middle cerebral artery was observed. Cysteine-altering *NOTCH3* variants linked to CADASIL have a prevalence of 3.4 individuals per 1000 in the global population, with a notably higher frequency of 9.0 individuals per 1000 in East Asia [103]. The *RNF213* p.Arg4810Lys variant (c.14429G > A) is present in approximately 1.5% of healthy individuals in Japan and Korea, 0.5% in China, and 80% to 90% of familial moyamoya disease cases in Japan and Korea, as well as 20% to 30% of familial cases in China [12]. Conversely, this variant is rarely found in individuals of White descent, with an allele frequency of less than 0.0006. Distinct *RNF213* variants, such as p.Asn4013Asp and p.Arg4062Glu, have been documented in White patients with moyamoya disease [12]. Moreover, the *RNF213* p.Arg4810Lys variant is prevalent among patients with large artery atherosclerosis and is considered a significant risk factor for intracranial artery stenosis [104]. Given the elevated frequencies of the *NOTCH3* and *RNF213* variants in East Asia, it is estimated that the occurrence of double heterozygous variants for these two genes is about 1 in 7000 individuals in that region. Notably, the *RNF213* p.Arg4810Lys variant carriers were identified in 6 out of 124 (4.8%) Japanese patients with CADASIL, indicating a significantly higher prevalence than the 1.5% found in the general population. The combined presence of both *NOTCH3* and *RNF213* variants may lead to a diagnosis of CADASIL due to an accelerated clinical course or a mixed phenotype [29]. The authors suggested that the early-onset ischemic strokes observed in this case might be due to this blended phenotype, although further analysis of additional cases is necessary to confirm this hypothesis.

## 4. Discussion

The review presented provides a comprehensive overview of the involvement of intracranial large arteries in CADASIL, particularly highlighting the association between CADASIL and intracranial stenosis, especially in Asian populations. The findings suggest that while CADASIL primarily manifests as a small vessel disease, there is a growing body of evidence indicating that larger vessels can also be affected, leading to significant clinical implications.

One of the strengths of the review is its critical interpretation of the existing literature, which combines clinical findings with genetic studies. The identification of the *RNF213* gene as a potential contributor to intracranial stenosis in CADASIL patients adds a crucial layer to our understanding of the disease’s pathophysiology [29,54]. Importantly, the review emphasizes that the observed association between CADASIL and large artery disease might not merely be coincidental, especially given the higher prevalence of *NOTCH3* mutations among Asian populations, which could predispose them to vascular abnormalities [25,56].

However, the limitations of the current understanding of CADASIL must also be acknowledged. The variability in clinical presentations and the challenges in establishing genotype–phenotype correlations underscore the complexity of this condition [3,8,11]. The review rightly points out that while some patients exhibit significant stenosis, others may not show any obvious large vessel involvement, raising questions about the underlying mechanisms. Furthermore, the role of traditional vascular risk factors, such as hypertension and diabetes mellitus, which are prevalent in the Asian population, complicates the interpretation of the data [60,62]. It remains to be clarified whether the observed stenosis is a direct result of CADASIL pathology or if it is exacerbated by these risk factors. In addition, the role of *RNF213* polymorphisms has to be considered, being highly prevalent in the Asian population. In fact, although the role of *NOTCH3* mutations has been suggested as causal in case-control studies on the same population, the simultaneous occurrence of *NOTCH3* mutations and *RNF213* polymorphism may be incidental.

Future perspectives on this topic should focus on longitudinal studies that aim to elucidate the relationship between CADASIL and large vessel involvement. Specifically, investigating whether the presence of *RNF213* polymorphisms influences the progression of large artery disease in CADASIL patients could provide valuable insights [29]. Additionally, understanding the potential role of atherosclerosis in this context is essential, as it may serve as a confounding factor in the observed associations [55]. Another source of variability is the potential association of multiple mutations in genes involved in small and large vessel diseases, determining a mixed and composite vasculopathy with variable phenotypes. However, we cannot exclude that the selective effect of *NOTCH3* mutations in a subset of patients harboring *RNF213* mutations is to enhance intracranial atherosclerosis [105], as described for the *ABCC6* gene [106]. A relevant issue is provided by the Asian prevalence of *RNF213* variants and related vasculopathy, the association in non-Asian populations being weaker but also less studied outside definite diseases (e.g., moyamoya arteriopathy). In fact, genetic testing for the *RNF213* variants is not recommended for Western moyamoya patients [107]. The exact functions, pathways, and biological relevance of *RNF213* are mostly unknown, and further research may identify pathways that could be the target of developing drugs for regulating angiogenesis and vascular remodeling. Finally, the role of epigenetic factors in the expression of both genes and their association should be considered, particularly in the occurrence of *RNF213*-associated vasculopathies.

## 5. Conclusions

In summary, CADASIL is primarily recognized as a small vessel disease, but emerging evidence supports the involvement of larger intracranial arteries, particularly in Asian populations. The potential association with *RNF213* polymorphisms raises intriguing questions regarding the genetic basis of this involvement and the potential role of epigenetic factors in Asian and non-Asian populations. However, the variability in clinical manifestations and the impact of traditional vascular risk factors highlight the need for further investigation. Understanding these relationships will be crucial for improving diagnostic and therapeutic strategies for CADASIL patients. In addition, the role of *RNF213* in intracranial and systemic vasculopathy has been established, but the biological pathways have not been fully elucidated in order to better understand its role in angiogenesis and inflammation. This step is crucial for the future development of a therapy able to counteract the thickening of the arterial wall in a pre-stenotic phase.

## Figures and Tables

**Table 1:** Main *RNF213* variants associated with intracranial stenosis in CADASIL patients.

*RNF213* Variant	Reference	Cases (n)	Controls (n)	Country	Main Findings
c.14576G > A	29	124	384	Japan	Carrier rate in CADASIL patients with intracranial stenosis: 23.5% (4/17) vs. 1.9% (2/107) without stenosis (*p* = 0.0032). Higher frequency of territorial infarction in carriers (75.0% vs. 20.0%, *p* = 0.0410). 2.6% of control population identified as carriers of the c.14576G > A variant. 21.9% of sporadic intracranial stenosis patients exhibited this variant in the Japanese population [5].
c.14429G > A (p.Arg4810Lys)	29	124	384	Japan	Found in 4.8% (6/124) of CADASIL patients; higher than 1.5% in the general population. Present in 80–90% of familial moyamoya disease cases; significant risk factor for intracranial artery stenosis [12].

## Data Availability

No new data were produced in this paper.
